# Clinical Utility of a Coronary Heart Disease Risk Prediction Gene Score in UK Healthy Middle Aged Men and in the Pakistani Population

**DOI:** 10.1371/journal.pone.0130754

**Published:** 2015-07-02

**Authors:** Katherine E. Beaney, Jackie A. Cooper, Saleem Ullah Shahid, Waqas Ahmed, Raheel Qamar, Fotios Drenos, Martin A. Crockard, Steve E. Humphries

**Affiliations:** 1 Centre for Cardiovascular Genetics, British Heart Foundation Laboratories, Institute of Cardiovascular Science, University College London, University Street, London, United Kingdom; 2 Department of Microbiology and Molecular Genetics, New Campus, University of the Punjab, Lahore, Pakistan; 3 Department of Microbiology, University of Haripur, Haripur, Pakistan; 4 COMSATS Institute of Information Technology, Park Road, Chak Shahzad, Islamabad, Pakistan; 5 Integrative Epidemiology Unit, School of Social and Community Medicine, University of Bristol, Bristol, United Kingdom; 6 Molecular Diagnostics Group, Randox Laboratories Ltd, Crumlin, United Kingdom; University of Tampere, FINLAND

## Abstract

**Background:**

Numerous risk prediction algorithms based on conventional risk factors for Coronary Heart Disease (CHD) are available but provide only modest discrimination. The inclusion of genetic information may improve clinical utility.

**Methods:**

We tested the use of two gene scores (GS) in the prospective second Northwick Park Heart Study (NPHSII) of 2775 healthy UK men (284 cases), and Pakistani case-control studies from Islamabad/Rawalpindi (321 cases/228 controls) and Lahore (414 cases/219 controls). The 19-SNP GS included SNPs in loci identified by GWAS and candidate gene studies, while the 13-SNP GS only included SNPs in loci identified by the CARDIoGRAMplusC4D consortium.

**Results:**

In NPHSII, the mean of both gene scores was higher in those who went on to develop CHD over 13.5 years of follow-up (19-SNP p=0.01, 13-SNP p=7x10^-3^). In combination with the Framingham algorithm the GSs appeared to show improvement in discrimination (increase in area under the ROC curve, 19-SNP p=0.48, 13-SNP p=0.82) and risk classification (net reclassification improvement (NRI), 19-SNP p=0.28, 13-SNP p=0.42) compared to the Framingham algorithm alone, but these were not statistically significant. When considering only individuals who moved up a risk category with inclusion of the GS, the improvement in risk classification was statistically significant (19-SNP p=0.01, 13-SNP p=0.04). In the Pakistani samples, risk allele frequencies were significantly lower compared to NPHSII for 13/19 SNPs. In the Islamabad study, the mean gene score was higher in cases than controls only for the 13-SNP GS (2.24 v 2.34, p=0.04). There was no association with CHD and either score in the Lahore study.

**Conclusion:**

The performance of both GSs showed potential clinical utility in European men but much less utility in subjects from Pakistan, suggesting that a different set of risk loci or SNPs may be required for risk prediction in the South Asian population.

## Introduction

Despite being largely preventable, coronary heart disease (CHD) remains the most common cause of the death worldwide [1, 2]. There is a growing CHD burden in the developing world [3], particularly in South Asian countries such as Pakistan where the prevalence of CHD in urban Karachi has approximately doubled since 1970[4]. This increase is most likely due to the adoption of a more “Western” lifestyle combined with a greater susceptibility to metabolic syndrome[5]. Many CHD risk factors, such as smoking status and hypertension, can be modified through lifestyle changes or drug therapy[6], which can greatly reduce CHD morbidity and mortality[7, 8]. Given that the atherosclerotic process can begin many decades before clinical manifestation, this provides an opportunity for preventative measures to be employed to avoid its escalation[9].

In order to target lifestyle and therapeutic intervention appropriately, those most at risk of developing CHD must be identified. A number of conventional risk factor (CRF) scores to determine 10-year risk CHD risk have been developed. These include the Framingham risk score [10], SCORE [11] and QRISK2 [12]. Individuals are then classified on the basis of the CRF score. Until recently the cut-off for the high risk category (those who qualify for statin treatment for primary prevention of CHD) was set at 20% ten year risk of CHD[6]. However, lower cut-offs have been proposed in both the USA [13] and the UK, with the 2014 National Institute for Health and Care Excellence (NICE) guidelines setting the threshold at 10%[14]. To date, such risk scores have provided only modest discrimination. Most events occur in individuals classified as being at intermediate risk [1, 15]. By improving risk prediction tools, preventative measures can be targeted more appropriately.

It is has been estimated that heritability of CHD is 40–50% [16] and more than forty CHD associated loci have been identified using both traditional candidate gene studies and genome-wide association studies (GWAS)[17, 18]. The most recent publication from the CARDIoGRAMplusC4D consortium [18] increased the number of robustly associated loci to 46. The inclusion of genetic information is therefore a good candidate to improve CHD risk prediction. As each variant is associated with a modest effect size, the addition of a single CHD risk variant to a CRF score does not improve risk stratification [19–21]. However, combining even a small number of variants into a “gene score” has been shown to improve classification or discrimination of individuals [22, 23].

The objective of this study was to determine whether the inclusion of a CHD risk gene score has clinical utility in the European population. We sought to do this by investigating whether addition of a gene score improves discrimination and prediction over and above the use of the Framingham CRF score alone in a prospective study of middle-aged men from the UK. We then looked to determine if the same genetic variants can be used in individuals from the Indian Subcontinent. Two gene scores, one with 19 SNPs taken from both GWAS and candidate gene studies and one with 13 SNPs where only SNPs in loci identified by the CARDIoGRAMplusC4D consortium were included, were tested.

## Methods

### NPHSII

NPHSII is a prospective CHD study of approximately 3000 men as described previously [2]. Briefly, middle-aged men (50–64) were recruited from 9 general practices in the UK. Anyone with a history of CHD or diabetes was excluded. There was a median of 13.5 years follow-up. CHD was defined as acute myocardial infarction (MI), silent MI or undergoing coronary surgery. All subjects gave written informed consent and the study had ethical approval from the national research ethics service (NRES) Committee London-Central.

### Pakistani cohorts

Two case-control studies from Pakistan were collected. One group was collected from the Rawalpindi Institute of Cardiology, Pakistan. All cases had had an MI as defined by a positive test for troponin T, ST segment changes on electrocardiogram and typical chest pain radiating in the chest that was not relieved at rest. This study will hereafter be referred to as the “Islamabad” study. The study had approval from the Institutional Review Board and Ethics Committee of Shifa College of Medicine, Shifa International Hospital, Islamabad and all subjects gave written informed consent. The second group were collected from hospitals in Lahore, Pakistan. All cases had CHD as defined by echocardiogram or angiography. This study will hereafter be referred to as the “Lahore” study. All subjects gave written informed consent and the study had ethical approval from the institutional ethical committee, University of the Punjab, Lahore. In both studies controls were taken from the general population.

### Gene Scores

The SNPs included in the gene scores are presented in Table 1, along with the source publication(s). Each SNP was weighted by its effect size as reported in their respective publications and the gene scores were calculated by multiplying the number of risk alleles by the natural log of the odds ratio for each SNP and adding the products together. It was assumed that all SNPs were acting in an additive manner apart from rs1799983, which was treated in a recessive manner. A 13 SNP score was also considered using only SNPs in loci identified by the most recent publication from the CARDIoGRAMplusC4D consortium [18].

**Table 1:** SNPs included in the gene scores.

Gene/Locus	SNP	SNP Location	Risk Allele	Odds Ratio	Reference
MIA3*	rs17465637	Intergenic	C	1.14	Kathiresan et al. [36]
9p21*	rs10757274	Intergenic	G	1.29	Kathiresan et al. [36]
*DAB2IP*	rs7025486	Intergenic	A	1.16	Harrison, Cooper [37]
CXCL12*	rs1746048	Intergenic	C	1.17	Kathiresan et al. [36], Samani, Erdmann [38]
*ACE*	rs4341	Intergenic	G	1.22	Casas, Cooper [17]
*NOS3*	rs1799983	E289D	T	1.31	Casas, Cooper [17]
APOA5*	rs662799	Promoter Variant	G	1.19	Sarwar, Sandhu [39]
*SMAD3*	rs17228212	Intergenic	C	1.21	Samani, Erdmann [38]
APOB*	rs1042031	E4181K	A	1.73	Casas, Cooper [17]
*CETP*	rs708272	Intronic	C	1.28	Casas, Cooper [17]
LPA*	rs3798220	I1891M	C	1.92	Clarke, Peden [40]
LPA*	rs10455872	Intergenic	G	1.70	Clarke, Peden [40]
MRAS*	rs9818870	Intergenic	T	1.15	Erdmann, Grosshennig [41]
LPL*	rs328	S447X	C	1.25	Casas, Cooper [17]
*LPL*	rs1801177	D9N	A	1.33	Sagoo, Tatt [42]
SORT1* ^+^	rs646776^+^	Intergenic	A	1.19	Kathiresan et al. [36]
PCSK9*	rs11591147	R46L	G	1.43	Benn, Nordestgaard [43]
APOE*	rs429358	C112R	C	1.06	Bennet, Di Angelantonio [44]
APOE*	rs7412	C158R	T^++^	0.80	Bennet, Di Angelantonio [44]

SNPs marked with an asterisk (*) are included in both the 19 and 13 SNP gene score.

^+^rs599839 was genotyped instead of rs646776, r^2^ = 0.95 in Europeans

^++^For rs7412, the protective SNP is included in the gene score

### Statistical analysis

Analysis for NPHSII was conducted using STATA v13.1[24]. Analysis for the Islamabad study and genotype frequency comparisons between all studies were conducted using *R* v3.0.3 [25]. Analysis for the Lahore study was conducted using SPSS version 22.0[26]. Variables were compared between the CHD and no-CHD groups using two-sided t-tests for continuous variables and χ^2^ tests for categorical variables. Hardy Weinberg equilibrium was assessed using χ^2^ tests. The Framingham 10-year risk score was calculated using the equations described in [10]. The relationship between quintile of gene score and CHD was investigated using logistic regression.

## Results

### Results from NPHSII

The baseline characteristics for the participants of NPHSII are presented in Table 2. As expected, the men who went on to develop CHD were older, had higher BMI, higher blood pressure, higher cholesterol, higher triglycerides and a higher proportion were smokers, at baseline. Moreover, those who went on to develop CHD had a statistically significantly higher 10 year CHD risk as calculated using the Framingham score.

**Table 2:** Baseline Characteristics of NPHSII participants.

Trait	NPHSIINo CHD(n = 2491)	NPHSIICHD(n = 284)	p-value
Age (years)	56.0 (3.4)	56.6 (3.5)	7x10^-3^
Male (%)	100	100	-
Smoking (%)	27	37.0	5x10^-4^
BMI (kg/m^2^)	26.4 (3.5)	26.9 (3.4)	0.04
Systolic Blood Pressure (mmHg)	137.9 (19.1)	142.5 (19.4)	2x10^-4^
Diastolic Blood Pressure (mmHg)	84.3 (11.2)	86.7 (11.4)	9x10^-4^
Cholesterol (mmol/l)	5.70 (1.01)	6.06 (1.02)	<1x10^-4^
Triglyceride (mmol/l)	1.77 (0.93)	2.05 (1.06)	<1x10^-4^
Framingham 10 year CHD risk	0.10 (0.07–0.15)	0.14 (0.09–0.20)	9x10^-4^
19 SNP Gene Score	3.30 (0.57)	3.42 (0.55)	0.01
13 SNP Gene Score	2.43 (0.48)	2.53 (0.45)	7x10^-3^

All variable are present as the mean plus standard deviation, apart from the Framingham 10 year CHD risk score where the mean and interquartile range are given.

The genotype frequency of each SNP in NPHSII is shown in S1 Table. All SNPs except rs1042031, in *APOB*, were in Hardy-Weinberg equilibrium. A comparison of risk allele frequency in those who did and those who did not go on to develop CHD is presented in S4 Table. There was a statistically significant difference between the two groups for rs10757274 at the 9p21 locus and rs1746048 (close to gene *CXCL12*), with the risk allele frequency being higher in the CHD group for both SNPs.

The genotype information was combined using a weighted genetic risk score. For the 19 SNP GS, full data for Framingham score plus gene score was available for 1164 individuals, 114 of whom developed CHD. For the 13 SNP GS full data for Framingham score plus gene score was available for 1437 individuals, 143 of whom developed CHD. Both gene scores were higher in those who developed CHD during follow-up (19 SNP GS p = 0.01, 13 SNP GS p = 7x10^-3^).

We examined the ability of the two gene scores to reclassify those who did and those who did not go on to develop CHD. As shown in Table 3, although more participants appeared to be reclassified correctly with the inclusion of both the 19 SNP and 13 SNP GS compared to the Framingham score alone, this increase was not statistically significant in either case. When only those who were reclassified into the high risk category (set at 10% ten-year risk) were considered (those with a strong genetic predisposition to CHD with intermediate and low CRF scores), a statistically significant number of participants were reclassified correctly (Table 3). This was also the case when the cut-off for high risk was set at 20% (shown in S5 Table).

**Table 3:** Reclassification of NPHSII participants with the addition of the gene scores to the Framingham conventional risk factor score.

Gene Score	CHD Status	Framingham10 year CHD risk	Reclassification: Increased Risk	Reclassification: Decreased Risk	Proportion Increased Risk
19 SNP	No CHD	<10%	103	132	22.9%
19 SNP	CHD	≥ 10%	11	9	45.8%
			Overall NRI: 4.5% (-3.7%-12.7%) p = 0.28		Increased risk p = 0.01
13 SNP	No CHD	<10%	115	145	20.0%
13 SNP	CHD	≥ 10%	13	12	34.2%
			Overall NRI: 3.0% (-4.3%-10.3%) p = 0.42		Increased risk p = 0.04

NRI = net reclassification index.

The predicative ability of using the Framingham CRF score was compared to the Framingham score plus the genetic risk score. There was no statistically significant increase in the area under the ROC curve for either the 19 SNP GS (p = 0.48) or the 13 SNP GS (p = 0.82) (S1 Fig).

**Fig 1:**
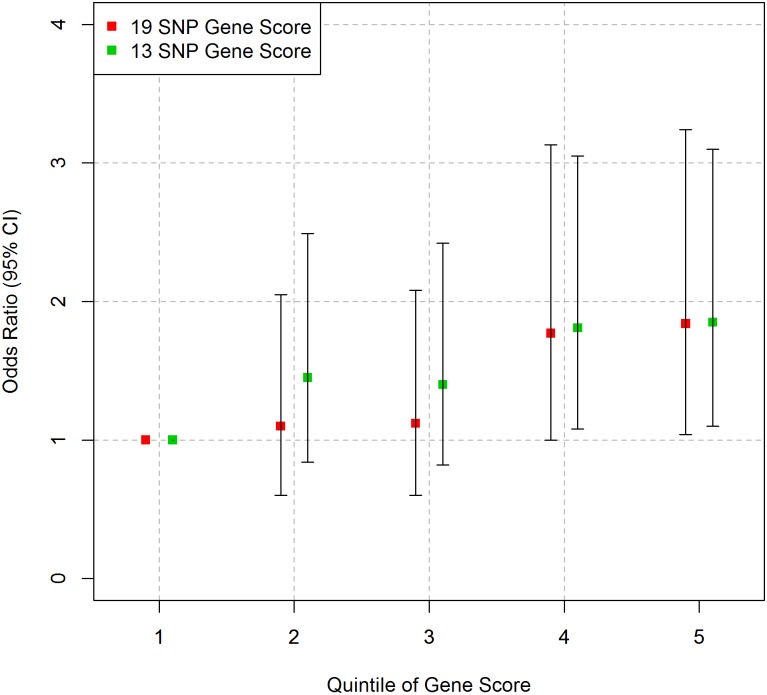
Association between gene score and CHD in NPHSII. Logistic regression (age adjusted) was performed for each group. Error bars represent 95% confidence intervals.

As shown in Fig 1 and S6 Table, there was a statistically significant trend for higher quintiles of gene score to have higher odds ratio for CHD compared to the bottom quintile for both the 19 SNP GS (p = 8x10^-3^) and the 13 SNP GS (p = 0.01) after adjustment for age.

### Results from Pakistani studies

We then attempted to transpose these scores in two case-control samples from Pakistan. The available conventional risk factor information for both studies is presented in Table 4. Data was missing for up to 60% of participants for some variables in the Islamabad study. As expected, in both studies the case group was older and had a higher proportion with diabetes, hypertension and smokers. There was no difference in the proportion of males present in each group. Surprisingly, in the Islamabad study, mean LDL cholesterol was not statistically significantly different between cases and controls. This can be attributed to the treatment of those post-MI with lipid lowering therapies. BMI, total cholesterol, triglycerides, family history and HDL cholesterol data were available for some of the participants of the Islamabad study. Only for HDL cholesterol was the difference between the two groups statistically significant, being lower in the cases.

**Table 4:** Characteristics of the Pakistani sample sets.

	Islamabad			Lahore		
Trait	ControlsN = 228	CasesN = 321	p-value	ControlsN = 219	CasesN = 404	p-value
Age (mean)	38(11.83)	53(11.80)	<2.2x10^-16^	56 (10.50)	59 (12.60)	2x10^-3^
Gender (% Female)	34%	31%	0.52	46%	41%	0.27
BMI (kg/m^2^)	24.2(3.95)	24.3(4.08)	0.85	-	-	-
TC (mmol/l)	4.52(1.38)	4.71(3.77)	0.56	-	-	-
LDL-C (mmol/l)	2.66(0.78)	2.55(0.99)	0.33	2.19 (0.44)	2.74 (0.75)	6.50x10^-22^
HDL-C (mmol/l)	1.35(1.01)	0.96(0.22)	1.01x10^-4^	-	-	-
TG (mmol /l)*	1.44 (0.91)	1.58 (1.06)	0.22	-	-	-
Diabetes (%)	1	32	6.6x10^-9^	14	65	5.10x10^-34^
Hypertension (%)	15	46	4.9x10^-7^	16	62	9.00x10^-28^
Family History (%)	42	42	0.96	-	-	-
Smoking (%)	25	46	1.25x10^-3^	11	30	4.30x10^-8^
19 SNP Gene Score	2.89 (0.50)	2.94(0.50)	0.35	2.92(0.53)	2.93(0.50)	0.75
13 SNP Gene Score	2.24(0.42)	2.34(0.42)	0.04	2.21(0.39)	2.21(0.39)	0.95

Categorical variables were compared using a χ^2^ test while continuous variables were compared using Welch’s t-tests.

* Log transformed data. Geometric mean and approximate SD are given.

The 19 SNPs were genotyped in both Pakistani sample sets and the results are presented in S1 Table. For the Islamabad study, five SNPs were not in Hardy-Weinberg equilibrium—*MIA3* rs17465637, *CXCL12* rs1746048, *MRAS* rs9818870, *LPL* rs1801177 and *SMAD3* rs17228212-with an excess of homozygotes. Genotypes were confirmed by sequencing. In the Lahore study, only one SNP–*LPA* rs10455872- was not in Hardy Weinberg equilibrium. The risk allele frequencies did not differ between the two Pakistani groups as shown in Table 5. The data from the control groups was combined and compared to that from the group in NPHSII. The risk allele frequency was lower in the Pakistani group for 13 SNPs and higher for three SNPs compared to NPHSII.

**Table 5:** Comparison of risk allele frequencies between the control groups from the Pakistani studies and between the combined total of the Pakistani control groups and NPHSII.

Gene/Locus	SNP	RAF Islamabad Controls	RAF Lahore Controls	p-value	RAF NPSHII(95% CI)	p-value
*MIA3*	rs17465367	0.64(0.59–0.69)	0.63(0.58–0.67)	0.68	0.71(0.69–0.72)	3.42x10^-5^
9p21	rs10757274	0.44(0.40–0.49)	0.46(0.42–0.51)	0.60	0.48(0.47–0.50)	3.16x10^-3^
*DAB2IP*	rs7025486	0.31 (0.26–0.35)	0.32(0.27–0.36)	0.84	0.26(0.17–0.24)	4.70x10^-4^
*CXCL12*	rs1746048	0.65 (0.61–0.70)	0.64(0.59–0.68)	0.65	0.86(0.85–0.87)	<2.20x10^-16^
*SMAD3*	rs17228212	0.19 (0.15–0.22)	0.18(0.14–0.21)	0.76	0.31(0.30–0.32)	4.88x10^-14^
*MRAS*	rs9818870	0.10 (0.07–0.13)	0.09(0.06–0.12)	0.75	0.16(0.15–0.17)	2.61x10^-6^
*SORT1*	rs599839	0.72 (0.68–0.77)	0.74(0.70–0.79)	0.55	0.78(0.77–0.79)	2.03x10^-3^
*ACE*	rs4341	0.41 (0.36–0.45)	0.47(0.43–0.52)	0.05	0.52(0.50–0.53)	3.09x10^-5^
*NOS3*	rs1799983	0.16 (0.13–0.20)	0.18(0.15–0.22)	0.51	0.33(0.32–0.35)	<2.20x10^-16^
APOA5	rs662799	0.15 (0.12–0.18)	0.17(0.13–0.20)	0.60	0.06(0.05–0.07)	<2.20x10^-16^
*APOB*	rs1042031	0.15 (0.12–0.19)	0.13(0.09–0.16)	0.24	0.18(0.17–0.19)	9.75x10^-3^
*CETP*	rs708272	0.55 (0.50–0.60)	0.56(0.51–0.61)	0.83	0.56(0.55–0.58)	0.62
*LPA*	rs3789220	0.01 (0–0.02)	0.003(0–0.01)	0.81	0.02(0.01–0.02)	0.02
*LPA*	rs10455872	0.01 (0–0.03)	0.01(0.00–0.03)	1	0.07(0.07–0.08)	1.06x10^-10^
*PCSK9*	rs11591147	1.00	0.995 (0.00–0.01)	0.50	0.99 (0.99–0.99)	0.07
*APOE*	rs429358	0.09 (0.06–0.11)	0.11 (0.08–0.14)	0.52	0.17 (0.16–0.18)	1.60x10^-9^
*APOE*	rs7412	0.96 (0.94–0.98)	0.96 (0.94–0.98)	1	0.91 (0.90–0.92)	2.69x10^-6^
*LPL*	rs328	0.92 (0.89–0.94)	0.91 (0.89–0.94)	0.98	0.90 (0.89–0.91)	0.12
*LPL*	rs1801177	0.01 (0–0.02)	0	0.14	0.01 (0.01–0.02)	0.04

Comparisons were performed using tests of proportion. RAF = Risk Allele Frequency, CI = Confidence Interval.

The 19 and 13 SNP GSs were calculated for both Pakistani studies and the results are shown in Table 4. For the 19 SNP GS, full genotyping was available for 304 samples (119 controls/175 cases) in the Islamabad study and for 438 samples (130 controls/308 cases) in the Lahore study. For the 13 SNP GS, full genotyping was available for 317 samples (123 controls/194 cases) in the Islamabad study and for 490 samples (145 controls/345 cases) in the Lahore study. In the Islamabad sample, a statistically significant increase in the mean gene score was observed between cases and controls for the 13 SNP GS (p = 0.04), but not for the 19 SNP GS (p = 0.35). For the Lahore sample, the mean gene score was not statistically significantly different between cases and controls for either score (19 SNP GS p = 0.75, 13 SNP GS p = 0.95). The 19 and 13 SNP GSs were found to be *lower* in the controls compared to those in NPHSII who did not go on to develop CHD in both the Islamabad (19 SNP GS p = 2.92 x10^-14^, 13 SNP GS p = 5.62x10^-6^) and the Lahore cohorts (19 SNP GS p = 7.78 x10^-13^, 13 SNP GS p = 1.49x10^-9^).

**Fig 2:**
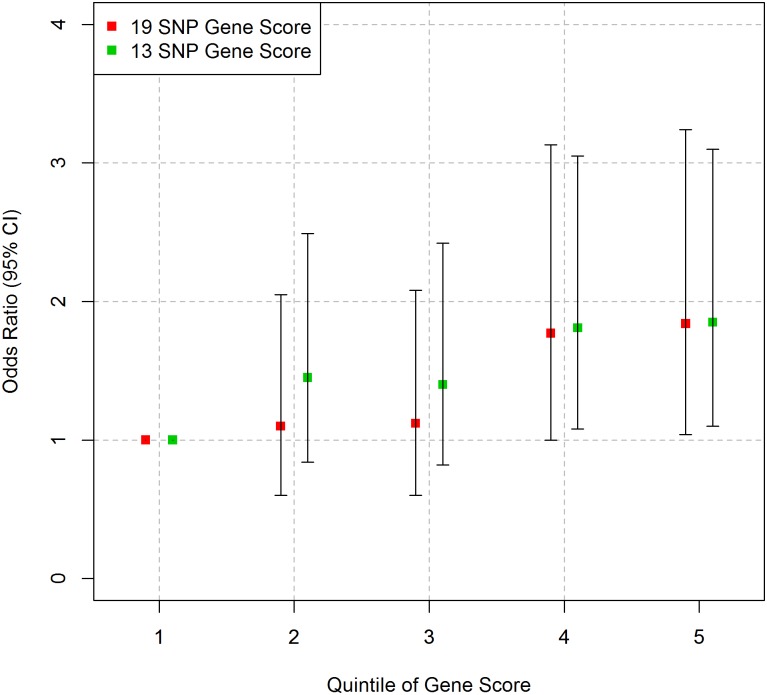
Association between gene score and outcome in the Pakistani samples. Logistic regression was performed for each group. (A) Islamabad study, outcome is MI, and (B) Lahore study, outcome is CHD. Error bars represent 95% confidence intervals.

While the higher quintiles of the 13 SNP GS gene score appear to have a greater odds ratio for outcome compared to the bottom quintile in the Islamabad sample, this was not statistically significant. No trend between quintile of gene score and outcome was observed for the 19 SNP GS in the Islamabad sample or for either GS in the Lahore sample (Fig 2 and S7 Table).

## Discussion

The aim of including genetic information in CHD risk prediction is to identify those who have a high overall risk but who would be classed as being at low or intermediate risk using conventional risk factor scores. In this study, we found that a 19 SNP GS, which uses CHD risk weighting from published meta-analyses, showed improved risk classification over and above the Framingham score alone in NPHSII when those men who went up a risk category (for example moving into the high risk category) were considered. The exclusion of six SNPs in non-CARDIoGRAMplusC4D loci had a modest detrimental effect on the utility of the gene score in the UK men, reducing the overall NRI non-significantly from 4.5% to 3.0% with a similar non-significant decrease in the number of men correctly reclassified into the high risk group. Thus the 19 SNP GS, appears to have the better utility. By contrast, in two case-control studies from Pakistan the gene scores did not perform as well. While in the subjects from Islamabad the 13 SNP GS showed a significant case-control effect, neither score showed utility in the samples from Lahore.

There are a number of potential reasons why the results from the European group were not confirmed in Pakistanis. Firstly both Pakistani studies comprise only a few hundred samples and thus sample size and statistical power is an issue. We estimated that should the same effect found in NPHSII be present in the Pakistani group, to have 80% power to detect it (at the 5% significance threshold), approximately 340 cases and approximately 340 controls would be required for both gene scores. This is well above the number of individuals for whom complete genotyping was available in both Pakistani studies. Secondly, “cases” were defined differently in the two Pakistani studies. There was very little difference between the mean gene score in cases and controls in the Lahore sample which used a much broader definition of CHD, compared to the Islamabad group which used an MI phenotype (more like the “hard” endpoints used in the prospective NPHSII). A further limitation of the study is that using retrospectively collected cases restricts the case group to those who have survived which may introduce a survivor bias to the sample.

A further possibility is SNP selection. While eight of the SNPs are non-synonymous (and thus are likely to be the functional), and the *APOA5* promoter variant has been shown to be directly functional [27] many are not in protein coding regions. It is possible that such SNPs merely “tag” the functional SNP located in the same linkage disequilibrium (LD) block. Given the differing LD patterns between ethnicities, it is possible that some of the SNPs used here are in strong LD with the functional SNP in Europeans but not in other ethnic groups. For example, the SNPs rs599839 and rs646776 are often used to tag each other at the *CELSR2-PSRC1-SORT1* locus, with rs599839 being known to be functional [28]. Data from the HapMap project [29] shows that the LD between the two SNPs is r^2^ = 0.95 in the Northern European group and r^2^ = 0.76 in the Japanese group but the two SNPs are not in LD in the Yoruba group (from Ibadan, Nigeria). However, a study of approximately 2000 Indians collected from Mumbai and Bangalore found the two SNPs to be in strong LD (r^2^ = 0.98) in this group. Furthermore, a number of SNPs at the 9p21 locus were genotyped in the PROMIS study of ethnic Pakistanis living in the UK and the LD pattern was found to be similar to Europeans at this locus [30] and a comparison of the genetic architecture of the *LPA* gene in individuals of different ethnicities (including South Asians) living in Canada found similar haplotype blocks to be present in the groups studied [31]. Therefore, for these loci we conclude that it is appropriate to use the SNP identified in studies with European participants. To what extent this can be extrapolated to the other SNPs and loci included is not clear, and for example, a study of *CETP* polymorphisms in the Punjabi population from Northern India found the LD pattern to differ from that observed in Europeans[32] but overall information regarding LD patterns in South Asians is limited. Moreover, given that the risk allele frequency is statistically significantly lower for 13 of the 19 SNPs in the Pakistani group compared to NPHSII, it is unsurprising that the gene scores are also statistically significantly lower. This shows that even if many of the SNPs are functional, then gene scores based on these SNPs will perform less well in the Pakistani group. In order to optimise the use of the gene scores for use in those of South Asian ethnicity, the functional SNP should be identified (bearing in mind that the functional SNP may differ between ethnic groups) in all cases to maximise the performance of a gene score based on this set of loci, and it may be necessary to design a South-Asian specific gene score.

We can only speculate whether the modest performance of the gene scores observed in Pakistani subjects here will be seen in samples from other parts of the Indian Subcontinent, as there is considerable genetic diversity within the region and since score performance is in part dependent on the frequency of the risk alleles. To date, allele frequency information across the Indian Subcontinent is limited for the SNPs used. A small number of Punjabi subjects (n = 96) from Lahore were genotyped as part of the 1000 genomes project (phase 3) [33]. No difference in allele frequencies was observed for any of the SNPs in this data set when compared with the control groups presented here. The risk allele frequency of the 9p21 SNPs genotyped in the PROMIS study were statistically significantly higher than observed here [30]. While a similar risk allele frequency to that found here was observed for rs662799 (in the *APOA5* promoter) in a Punjabi Pakistani case-control study, the risk allele frequency observed for rs599839 (at the *SORT1* locus) in that study was much lower than observed here [34]. Another factor which may influence how representative the results presented here are, is that five of the 19 SNPs genotyped in the Islamabad case-control study were not in Hardy-Weinberg equilibrium. In each case this was due to an excess of homozygotes compared to what would be expected. This is likely caused by the presence of population sub-structure[35]. Clearly much more data is required from larger samples representative of different regions and groups from the Indian Subcontinent to determine the frequencies of these SNPs.

Overall this work demonstrated that the use of a 19 SNP gene score has the potential to improve risk stratification in European individuals over and above classical CHD risk factors. Reducing the number of SNPs in the score to only the 13 with GWAS-proven effects on CHD risk had a modest detrimental effect on the utility. We do not have definitive evidence of the utility of either score in subjects from Pakistan and larger samples are required to determine this.

## Supporting Information

S1 Fig ROC curves for CHD prediction in NPHSII. (TIF)

S1 Table Risk allele frequency for each SNP in NPHSII and the Pakistani sample sets. HWE = Hardy Weinberg Equilibrium, CI = Confidence Interval.
(DOCX)

S2 Table Comparison of risk allele frequency between Lahore controls and NPHSII. Comparisons were performed using proportion tests. CI = Confidence Interval.
(DOCX)

S3 Table Comparison of risk allele frequency between Islamabad controls and NPHSII. Comparisons were performed using tests of proportion. CI = Confidence Interval.
(DOCX)

S4 Table Comparison of risk allele frequencies in those who did and did not develop CHD during follow-up of NPHSII. Comparisons were performed using proportion tests. CI = Confidence Interval.
(DOCX)

S5 Table Reclassification of NPHSII participants with the addition of the gene scores to the Framingham conventional risk factor score (20% cut-off for high risk). NRI = Net Reclassification Index.
(DOCX)

S6 Table Odds ratio for CHD in NPHSII by quintile of gene score, compared to the lowest quintile. Logistic regression, age adjusted, was performed for each group. OR = Odds Ratio, CI = Confidence Interval.
(DOCX)

S7 Table Odds ratio for outcome (MI for Islamabad, CHD for Lahore) by quintile of gene score, compared to the lowest quintile. Logistic regression, adjusted for age and sex, was performed for each group. OR = Odds Ratio, CI = Confidence Interval. For the Islamabad study full data was available for 258 participants for the 19 SNP GS (106 controls/152 cases) and 268 participants for the 13 SNP GS (110 controls/158 cases). For the Lahore study full data was available for 438 participants for the 19 SNP GS (130 controls/308 cases) and 490 participants for the 13 SNP GS (145 controls/345 cases).
(DOCX)
