# Correction: Clinical Utility of a Coronary Heart Disease Risk Prediction Gene Score in UK Healthy Middle Aged Men and in the Pakistani Population

**DOI:** 10.1371/journal.pone.0139651

**Published:** 2015-09-28

**Authors:** Katherine E. Beaney, Jackie A. Cooper, Saleem Ullah Shahid, Waqas Ahmed, Raheel Qamar, Fotios Drenos, Martin A. Crockard, Steve E. Humphries

The image for Fig 2 is incorrect. Please see the corrected Fig 2 here.

**Fig 2:**
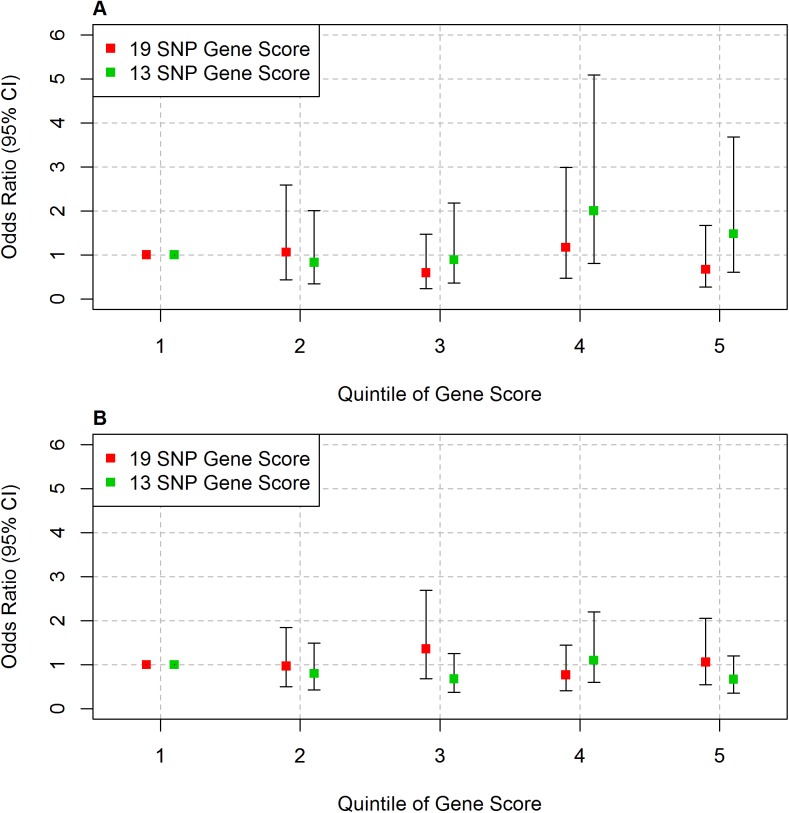
Association between gene score and outcome in the Pakistani samples. Logistic regression was performed for each group. (A) Islamabad study, outcome is MI, and (B) Lahore study, outcome is CHD. Error bars represent 95% confidence intervals.
