# Supplementary figures and images for: Clinical Utility of a Coronary Heart Disease Risk Prediction Gene Score in UK Healthy Middle Aged Men and in the Pakistani Population
Source: PLoS One. 2015 Jul 2;10(7):e0130754. doi: 10.1371/journal.pone.0130754 (PMC4489836; doi:10.1371/journal.pone.0130754)

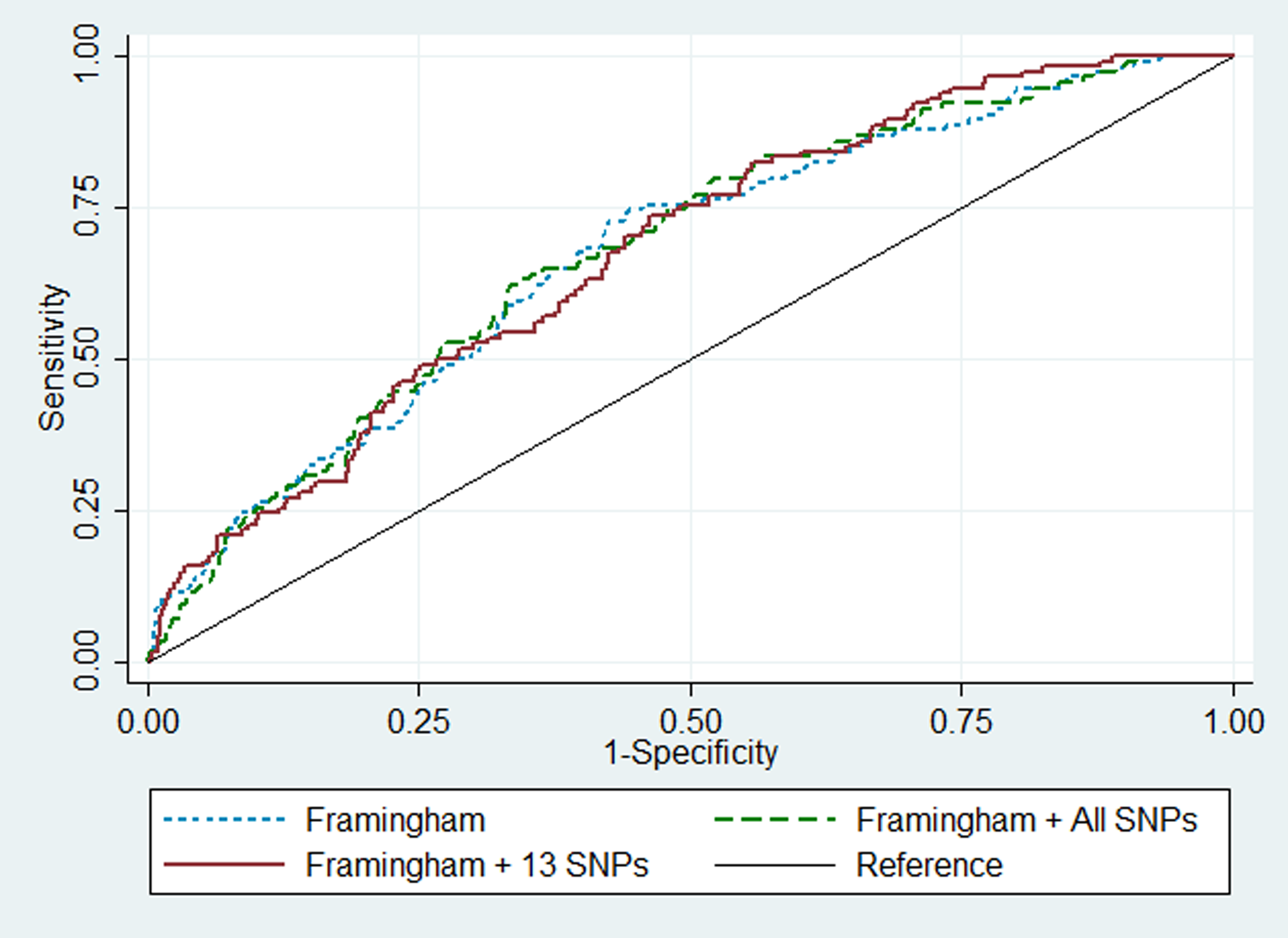

Supplement: S1 Fig — (TIF) [file pone.0130754.s001.tif]
