# Supplementary material for: Clinical Utility of a Coronary Heart Disease Risk Prediction Gene Score in UK Healthy Middle Aged Men and in the Pakistani Population
Source: PLoS One. 2015 Jul 2;10(7):e0130754. doi: 10.1371/journal.pone.0130754 (PMC4489836; doi:10.1371/journal.pone.0130754)
Supplement: S1 Table — HWE = Hardy Weinberg Equilibrium, CI = Confidence Interval. (DOCX) [file pone.0130754.s002.docx]

S1 Table: Risk allele frequency for each SNP in NPHSII and the Pakistani sample sets.

| Gene/Locus | SNP | NPHSII RAF  (95% CI) | P value HWE | Islamabad  RAF  (95% CI) | P value HWE | Lahore  RAF  (95% CI) | P value HWE |
| --- | --- | --- | --- | --- | --- | --- | --- |
| *MIA3* | rs17465367 | 0.71  (0.69-0.72) | 0.97 | 0.67  (0.63-0.69) | 0.01 | 0.64  (0.61-0.67) | 0.53 |
| 9p21 | rs10757274 | 0.48  (0.47-0.50) | 0.41 | 0.51  (0.48-0.54) | 0.09 | 0.49  (0.46-0.52) | 0.40 |
| *DAB2IP* | rs7025486 | 0.26  (0.17-0.24) | 0.28 | 0.30  (0.27-0.33) | 0.24 | 0.32  (0.29-0.34) | 0.80 |
| *CXCL12* | rs1746048 | 0.86  (0.85-0.87) | 0.85 | 0.69  (0.66-0.72) | 1.4x10^-3^ | 0.66  (0.64-0.69) | 0.94 |
| *SMAD3* | rs17228212 | 0.31  (0.30-0.32) | 0.09 | 0.19  (0.17-0.22) | 0.05 | 0.20  (0.17-0.22) | 0.85 |
| *MRAS* | rs9818870 | 0.16  (0.15-0.17) | 0.86 | 0.10  (0.09-0.12) | 0.03 | 0.09  (0.08-0.11) | 0.86 |
| *SORT1* | rs646776 | 0.78  (0.77-0.79) | 0.18 | 0.75  (0.72-0.77) | 0.76 | 0.75  (0.72-0.77) | 0.18 |
| *ACE* | rs4341 | 0.52  (0.50-0.53) | 0.32 | 0.42  (0.39-0.45) | 0.13 | 0.44  (0.41-0.47) | 0.16 |
| *NOS3* | rs1799983 | 0.33  (0.32-0.35) | 0.91 | 0.17  (0.15-0.19) | 0.06 | 0.19  (0.17-0.21) | 0.11 |
| APOA5 | rs662799 | 0.06  (0.05-0.07) | 0.15 | 0.16  (0.14-0.18) | 0.37 | 0.17  (0.14-0.19) | 0.99 |
| *APOB* | rs1042031 | 0.18  (0.17-0.19) | 0.04 | 0.14  (0.12-0.16) | 0.14 | 0.10  (0.08-0.11) | 0.85 |
| *CETP* | rs708272 | 0.56  (0.55-0.58) | 0.07 | 0.53  (0.50-0.56) | 0.70 | 0.55  (0.52-0.58) | 0.36 |
| *LPA* | rs3789220 | 0.02  (0.01-0.02) | 1.00 | 0.01  (0-0.01) | 0.87 | 0.003  (0.00-0.01) | 0.93 |
| *LPA* | rs10455872 | 0.07  (0.07-0.08) | 0.42 | 0.01  (0-0.02) | 0.82 | 0.02  (0.01-0.02) | 0.01 |
| *PCSK9* | rs11591147 | 0.99  (0.99-0.99) | 0.18 | 1 | - | 1.00  (0.99-1.00) | 0.95 |
| *APOE* | rs429358 | 0.17  (0.16-0.18) | 0.46 | 0.09  (0.07-0.11) | 0.03 | 0.11  (0.08-0.14) | 0.28 |
| *APOE* | rs7412 | 0.91  (0.90-0.92) | 0.85 | 0.96  (0.94-0.97) | 0.27 | 0.96  (0.94-0.98) | 0.33 |
| *LPL* | rs328 | 0.90  (0.89-0.94) | 0.77 | 0.92  (0.91-0.94) | 0.65 | 0.93  (0.91-0.94) | 0.64 |
| *LPL* | rs1801177 | 0.01  (0.01-0.02) | 0.08 | 4x10^-3^  (0-0.01) | <1x10^-4^ | 0 | - |

HWE=Hardy Weinberg Equilibrium, CI=Confidence Interval.
