# Supplementary material for: Clinical Utility of a Coronary Heart Disease Risk Prediction Gene Score in UK Healthy Middle Aged Men and in the Pakistani Population
Source: PLoS One. 2015 Jul 2;10(7):e0130754. doi: 10.1371/journal.pone.0130754 (PMC4489836; doi:10.1371/journal.pone.0130754)
Supplement: S2 Table — Comparisons were performed using proportion tests. CI = Confidence Interval. (DOCX) [file pone.0130754.s003.docx]

S2 Table: Comparison of risk allele frequency between Lahore controls and NPHSII.

| Gene/Locus | SNP | RAF Lahore Controls  (95% CI) | RAF NPHSII  (95% CI) | p-value |
| --- | --- | --- | --- | --- |
| *MIA3* | rs17465367 | 0.63  (0.58-0.67) | 0.71  (0.69-0.72) | 6.53 x10^-4^ |
| 9p21 | rs10757274 | 0.46  (0.42-0.51) | 0.48  (0.47-0.50) | 0.51 |
| *DAB2IP* | rs7025486 | 0.32  (0.27-0.36) | 0.26  (0.17-0.24) | 6.34 x10^-3^ |
| *CXCL12* | rs1746048 | 0.64  (0.59-0.68) | 0.86  (0.85-0.87) | <2.20 x10^-16^ |
| *SMAD3* | rs17228212 | 0.18  (0.14-0.21) | 0.31  (0.30-0.32) | 1.51 x10^--8^ |
| *MRAS* | rs9818870 | 0.09  (0.06-0.12) | 0.16  (0.15-0.17) | 2.79 x10^-4^ |
| *SORT1* | rs646776 | 0.74  (0.70-0.79) | 0.78  (0.77-0.79) | 0.10 |
| *ACE* | rs4341 | 0.48  (0.43-0.52) | 0.52  (0.50-0.53) | 0.10 |
| *NOS3* | rs1799983 | 0.18  (0.15-0.22) | 0.33  (0.32-0.35) | 2.64 x10^-10^ |
| *APOA5* | rs662799 | 0.17  (0.13-0.20) | 0.06  (0.05-0.07) | 5.61 x10^-16^ |
| *APOB* | rs1042301 | 0.13  (0.09-0.16) | 0.18  (0.17-0.19) | 7.87 x10^-3^ |
| *CETP* | rs708272 | 0.56  (0.51-0.61) | 0.56  (0.55-0.58) | 0.89 |
| *LPA* | rs3789220 | 0.003  (0.00-0.01) | 0.02  (0.01-0.02) | 0.05 |
| *LPA* | rs10455872 | 0.01  (0.00-0.03) | 0.07  (0.07-0.08) | 3.80 x10^-3^ |
| *PCSK9* | rs11591147 | 0.995  (0.00-0.01) | 0.99  (0.99-0.99) | 0.50 |
| *APOE* | rs429358 | 0.11  (0.08-0.14) | 0.17  (0.16-0.18) | 0.03 |
| *APOE* | rs7412 | 0.96  (0.94-0.98) | 0.91  (0.90-0.92)) | 4.53 x10^-4^ |
| *LPL* | rs328 | 0.91  (0.89-0.94) | 0.90  (0.89-0.94) | 0.34 |
| *LPL* | rs1801177 | 0 | 0.01  (0.01-0.02) | 0.03 |

Comparisons were performed using proportion tests. CI=Confidence Interval.
