# Supplementary material for: Clinical Utility of a Coronary Heart Disease Risk Prediction Gene Score in UK Healthy Middle Aged Men and in the Pakistani Population
Source: PLoS One. 2015 Jul 2;10(7):e0130754. doi: 10.1371/journal.pone.0130754 (PMC4489836; doi:10.1371/journal.pone.0130754)
Supplement: S3 Table — Comparisons were performed using tests of proportion. CI = Confidence Interval. (DOCX) [file pone.0130754.s004.docx]

S3 Table: Comparison of risk allele frequency between Islamabad controls and NPHSII.

| Gene/Locus | SNP | RAF Islamabad Controls  (95% CI) | RAF NPSHII  (95% CI) | p-value |
| --- | --- | --- | --- | --- |
| *MIA3* | rs17465367 | 0.64  (0.59-0.69) | 0.71  (0.69-0.72) | 9.10 x 10^-3^ |
| 9p21 | rs10757274 | 0.44  (0.40-0.49) | 0.48  (0.47-0.50) | 0.14 |
| *DAB2IP* | rs7025486 | 0.31  (0.26-0.35) | 0.26  (0.17-0.24) | 0.02 |
| *CXCL12* | rs1746048 | 0.65  (0.61-0.70) | 0.86  (0.85-0.87) | <2.20 x 10^-16^ |
| *SMAD3* | rs17228212 | 0.19  (0.15-0.22) | 0.31  (0.30-0.32) | 1.55 x 10^-7^ |
| *MRAS* | rs9818870 | 0.10  (0.07-0.13) | 0.16  (0.15-0.17) | 1.85 x 10^-3^ |
| *SORT1* | rs646776 | 0.72  (0.68-0.77) | 0.78  (0.77-0.79) | 5.65 x 10^-3^ |
| *ACE* | rs4341 | 0.41  (0.36-0.45) | 0.52  (0.50-0.53) | 1.20 x 10^-5^ |
| *NOS3* | rs1799983 | 0.16  (0.13-0.20) | 0.33  (0.32-0.35) | 8.05 x 10^-12^ |
| APOA5 | rs662799 | 0.15  (0.14-0.18) | 0.06  (0.05-0.07) | 1.15 x 10^-11^ |
| *APOB* | rs1042301 | 0.15  (0.12-0.18) | 0.18  (0.17-0.19) | 0.30 |
| *CETP* | rs708272 | 0.55  (0.50-0.60) | 0.56  (0.55-0.58) | 0.59 |
| *LPA* | rs3789220 | 0.01  (0.00-0.01) | 0.02  (0.01-0.02) | 0.27 |
| *LPA* | rs10455872 | 0.01  (0.00-0.03) | 0.07  (0.07-0.08) | 6.60 x 10^-6^ |
| *PCSK9* | rs11591147 | 1.00 | 0.99  (0.99-0.99) | 0.10 |
| *APOE* | rs429358 | 0.09  (0.06-0.12) | 0.17  (0.16-0.18) | 4.36 x 10^-6^ |
| *APOE* | rs7412 | 0.96  (0.94-0.98) | 0.91  (0.90-0.92) | 1.55 x 10^-3^ |
| *LPL* | rs328 | 0.92  (0.89-0.94) | 0.90  (0.89-0.91) | 0.21 |
| *LPL* | rs1801177 | 0.01  (0.00-0.02) | 0.01  (0.01-0.02) | 0.57 |

Comparisons were performed using tests of proportion. CI=Confidence Interval.
