# Supplementary material for: Clinical Utility of a Coronary Heart Disease Risk Prediction Gene Score in UK Healthy Middle Aged Men and in the Pakistani Population
Source: PLoS One. 2015 Jul 2;10(7):e0130754. doi: 10.1371/journal.pone.0130754 (PMC4489836; doi:10.1371/journal.pone.0130754)
Supplement: S4 Table — Comparisons were performed using proportion tests. CI = Confidence Interval. (DOCX) [file pone.0130754.s005.docx]

S4 Table: Comparison of risk allele frequencies in those who did and did not develop CHD during follow-up of NPHSII.

|  |  |  | NPHSII  No CHD | NPHSII  CHD |  |
| --- | --- | --- | --- | --- | --- |
| Gene/Locus | SNP | Risk allele | RAF (95% CI) | RAF (95% CI) | P value |
| *MIA3* | rs17465367 | C | 0.71  (0.69-0.728) | 0.71  (0.67-0.75) | 0.70 |
| 9p21 | rs10757274 | G | 0.48  (0.462-0.490) | 0.54  (0.50-0.58) | 6x10^-3^ |
| *DAB2IP* | rs7025486 | A | 0.25  (0.24-0.27) | 0.28  (0.24-0.32) | 0.16 |
| *CXCL12* | rs1746048 | C | 0.86  (0.85-0.87) | 0.89  (0.87-0.92) | 0.03 |
| *SMAD3* | rs17228212 | C | 0.31  (0.30-0.32) | 0.29  (0.26-0.33) | 0.42 |
| *MRAS* | rs9818870 | T | 0.15  (0.14-0.16) | 0.18  (0.15-0.21) | 0.16 |
| *SORT1* | rs646776 | A | 0.78  (0.77-0.79) | 0.79  (0.76-0.83) | 0.61 |
| *ACE* | rs4341 | G | 0.52  (0.50-0.53) | 0.52  (0.47-0.56) | 0.96 |
| *NOS3* | rs1799983 | T | 0.34  (0.32-0.35) | 0.31  (0.27-0.35) | 0.25 |
| APOA5 | rs662799 | G | 0.06  (0.05-0.07) | 0.06  (0.04-0.09) | 0.79 |
| *APOB* | rs1042301 | A | 0.18  (0.16-0.19) | 0.19  (0.15-0.22) | 0.71 |
| *CETP* | rs708272 | C | 0.57  (0.55-0.58) | 0.54  (0.50-0.59) | 0.31 |
| *LPA* | rs3789220 | C | 0.02  (0.01-0.02) | 0.02  (0.01-0.04) | 0.18 |
| *LPA* | rs10455872 | G | 0.07  (0.07-0.08) | 0.09  (0.07-0.12) | 0.11 |
| *PCSK9* | rs11591147 | G | 0.99  (0.99-0.99) | 0.996  (0.99-1.000) | 0.32 |
| *APOE* | rs429358 | C | 0.16  (0.15-0.18) | 0.18  (0.15-0.22) | 0.40 |
| *APOE* | rs7412 | C | 0.91  (0.90-0.91) | 0.93  (0.91-0.95) | 0.06 |
| *LPL* | rs328 | C | 0.90  (0.89-0.90) | 0.91  (0.88-0.93) | 0.26 |
| *LPL* | rs1801177 | A | 0.01  (0.01-0.02) | 0.02  (0.01-0.04) | 0.24 |

Comparisons were performed using proportion tests. CI=Confidence Interval.
