# Supplementary material for: Clinical Utility of a Coronary Heart Disease Risk Prediction Gene Score in UK Healthy Middle Aged Men and in the Pakistani Population
Source: PLoS One. 2015 Jul 2;10(7):e0130754. doi: 10.1371/journal.pone.0130754 (PMC4489836; doi:10.1371/journal.pone.0130754)
Supplement: S5 Table — NRI = Net Reclassification Index. (DOCX) [file pone.0130754.s006.docx]

S5 Table: Reclassification of NPHSII participants with the addition of the gene scores to the Framingham conventional risk factor score (20% cut-off for high risk).

| Gene Score | CHD Status | Framingham 10 year CHD risk | Reclassification:  Increased risk | Reclassification:  Decreased risk | Proportion Increased Risk |
| --- | --- | --- | --- | --- | --- |
| 19 SNP | No CHD | >=20% | 124 | 59 | 13.8 % |
| 19 SNP | CHD | <20% | 20 | 7 | 45.8 % |
|  | | | Overall NRI: 5.2 % (-4.1% - 14.5 %) p=0.27 | | Increased risk p=5x10^-3^ |
| 13 SNP | No CHD | >=20% | 130 | 55 | 11.6 % |
| 13 SNP | CHD | <20% | 21 | 11 | 20.2 % |
|  | | | Overall NRI: 1.2% (-6.8 % - 9.2 %) p=0.77 | | Increased risk p=0.01 |

NRI=Net Reclassification Index
