# Supplementary material for: Clinical Utility of a Coronary Heart Disease Risk Prediction Gene Score in UK Healthy Middle Aged Men and in the Pakistani Population
Source: PLoS One. 2015 Jul 2;10(7):e0130754. doi: 10.1371/journal.pone.0130754 (PMC4489836; doi:10.1371/journal.pone.0130754)
Supplement: S6 Table — Logistic regression, age adjusted, was performed for each group. OR = Odds Ratio, CI = Confidence Interval. (DOCX) [file pone.0130754.s007.docx]

S6 Table: Odds ratio for CHD in NPHSII by quintile of gene score, compared to the lowest quintile.

|  | 19 SNP GS | 13 SNP GS |
| --- | --- | --- |
| Quintile of  Gene Score | OR  (95% CI) | OR  (95% CI) |
| 1 | 1.00 | 1.00 |
| 2 | 1.20  (0.78-1.85) | 1.58  (1.00-2.50) |
| 3 | 1.39  (0.91-2.11) | 1.83  (1.17-2.85) |
| 4 | 1.61  (1.07-2.42) | 2.20  (1.42-3.40) |
| 5 | 1.43  (0.94-2.17) | 2.31  (1.50-3.56) |
| P value (trend) | p=8x10^-3^ | p=0.01 |

Logistic regression, age adjusted, was performed for each group. OR=Odds Ratio, CI=Confidence Interval.
