# Supplementary material for: Clinical Utility of a Coronary Heart Disease Risk Prediction Gene Score in UK Healthy Middle Aged Men and in the Pakistani Population
Source: PLoS One. 2015 Jul 2;10(7):e0130754. doi: 10.1371/journal.pone.0130754 (PMC4489836; doi:10.1371/journal.pone.0130754)
Supplement: S7 Table — Logistic regression, adjusted for age and sex, was performed for each group. OR = Odds Ratio, CI = Confidence Interval. For the Islamabad study full data was available for 258 participants for the 19 SNP GS (106 controls/152 cases) and 268 participants for the 13 SNP GS (110 controls/158 cases). For the Lahore study full data was available for 438 participants for the 19 SNP GS (130 controls/308 cases) and 490 participants for the 13 SNP GS (145 controls/345 cases). (DOCX) [file pone.0130754.s008.docx]

S7 Table: Odds ratio for outcome (MI for Islamabad, CHD for Lahore) by quintile of gene score, compared to the lowest quintile.

|  | 19 SNP GS-Islamabad | 13 SNP GS-Islamabad | 19 SNP GS-Lahore | 13 SNP GS- Lahore |
| --- | --- | --- | --- | --- |
| Quintile of  Gene Score | OR  (95%CI) | OR  (95%CI) | OR  (95%CI) | OR  (95%CI) |
| 1 | 1.00 | 1.00 | 1.00 | 1.00 |
| 2 | 1.06  (0.43-2.59) | 0.83  (0.34-2.00) | 0.97  (0.50-1.85) | 0.80  (0.43-1.49) |
| 3 | 0.59  (0.24-1.47) | 0.89  (0.35-2.17) | 1.36  (0.68-2.69) | 0.68  (0.37-1.26) |
| 4 | 1.17  (0.47-2.99) | 2.00  (0.81-5.09) | 0.77  (0.41-1.45) | 1.10  (0.60-2.20) |
| 5 | 0.67  (0.27-1.67) | 1.48  (0.60-3.68) | 1.06  (0.55-2.06) | 0.67  (0.36-1.20) |
| P value (trend) | 0.51 | 0.12 | 0.87 | 0.52 |

Logistic regression, adjusted for age and sex, was performed for each group. OR=Odds Ratio, CI=Confidence Interval. For the Islamabad study full data was available for 258 participants for the 19 SNP GS (106 controls/152 cases) and 268 participants for the 13 SNP GS (110 controls/158 cases). For the Lahore study full data was available for 438 participants for the 19 SNP GS (130 controls/308 cases) and 490 participants for the 13 SNP GS (145 controls/345 cases).
